# Bioinstructive Micro-Nanotextured Zirconia Ceramic Interfaces for Guiding and Stimulating an Osteogenic Response In Vitro

**DOI:** 10.3390/nano10122465

**Published:** 2020-12-09

**Authors:** Livia Elena Sima, Anca Bonciu, Madalina Baciu, Iulia Anghel, Luminita Nicoleta Dumitrescu, Laurentiu Rusen, Valentina Dinca

**Affiliations:** 1 Department of Molecular Cell Biology, Institute of Biochemistry, Romanian Academy, 296 Splaiul Independentei, 060031 Bucharest, Romania; lsima@biochim.ro; 2 Lasers Department, National Institute for Lasers, Plasma and Radiation Physics, 409 Atomistilor Street, 077125 Magurele, Romania; anca.bonciu@inflpr.ro (A.B.); iulia.anghel@inflpr.ro (I.A.); nicoleta.dumitrescu@inflpr.ro (L.N.D.); 3 Faculty of Physics, University of Bucharest, 077125 Magurele, Romania; 4 Otorhinolaryngology Department, MedLife Clinic, 79-81 Bvd. Tomis, 900178 Constanta, Romania; mcazan@medlife.ro; 5 IN2-FOTOPLASMAT Center, National Institute for Lasers, Plasma and Radiation Physics, 409 Atomistilor Street, 077125 Magurele, Romania

**Keywords:** Zirconia, mesenchymal stem cells, micro-nano topography, osteogenic differentiation, bone-anchored hearing aid (BAHA)

## Abstract

Osseous implantology’s material requirements include a lack of potential for inducing allergic disorders and providing both functional and esthetic features for the patient’s benefit. Despite being bioinert, Zirconia ceramics have become a candidate of interest to be used as an alternative to titanium dental and cochlear bone-anchored hearing aid (BAHA) implants, implying the need for endowing the surface with biologically instructive properties by changing basic parameters such as surface texture. Within this context, we propose anisotropic and isotropic patterns (linear microgroove arrays, and superimposed crossline microgroove arrays, respectively) textured in zirconia substrates, as bioinstructive interfaces to guide the cytoskeletal organization of human mesenchymal stem cells (hMSCs). The designed textured micro-nano interfaces with either steep ridges and microgratings or curved edges, and nanoroughened walls obtained by direct femtosecond laser texturing are used to evaluate the hMSC response parameters and osteogenic differentiation to each topography. Our results show parallel micro line anisotropic surfaces are able to guide cell growth only for the steep surfaces, while the curved ones reduce the initial response and show the lowest osteogenic response. An improved osteogenic phenotype of hMSCs is obtained when grown onto isotropic grid/pillar-like patterns, showing an improved cell coverage and Ca/P ratio, with direct implications for BAHA prosthetic development, or other future applications in regenerating bone defects.

## 1. Introduction

Implants are essential medical devices that provide structural support for diseased or destroyed organs. The biological processes occurring after implantation start with protein adsorption and end with new tissue formation at the body–implant interface [1,2,3]. The type of tissue formed at this interface is correlated to the implant bulk and surface characteristics, to the implantation site, and will further impact the long-term fate (biointegration or failure) of the implant [1]. An essential requirement for implant devices is the use of materials that provide key characteristics in terms of surface characteristics, mechanical properties and biointegration potential into the human body. The increased life span, resistance to fracture, mechanical strength, and good compatibility with human tissues of metal-based implants make Ti and its alloys the main candidates for dental and orthopedic implantology [4,5,6,7,8]. Coatings, mainly based on ceramics or hydroxylapatite (HA), aim at improving osteoconduction and have been used for clinical trial as an upgrade to the standard of care [9].

Nevertheless, the wear products in the tissue surrounding the implant due to corrosion stimulated by body fluids and the tissue microenvironment can cause periprosthetic organ/bone loss or allergies [10,11].

Based on its higher flexure strength, hydrothermal and mechanical resistance, high resistance to crack propagation, high fracture toughness (6.5–8 MPam1/2), inertness, low corrosion, lack of oncogenic effects, and higher affinity for bone tissue, Yttrium stabilized Tetragonal Zirconium-Zirconia also has gained special interest in the field of artificial limbs and dental joints [12,13,14,15,16,17,18]. There are already commercially available Zirconia ceramic implants for dentistry, nevertheless, its surface still needs improvement for enhanced biological response in terms of osseointegration, bone-to-implant interface strength, and resistance to long-term functional loading. The surface modification could represent an alternative strategic solution to reduce the chronic or excessive inflammation response, and fibrous encapsulation, respectively, for other types of implants as well, such as bone-anchored hearing aid (BAHA) implants.

It is already known that cell responsivity (i.e., cell adhesion, migration, cytoskeletal organization, gene expression regulation, and even differentiation) is directly correlated not only to the chemical composition of the environment but also to its stiffness, shape, and topography [19,20]. Research in recent years revealed that combined nano- and microscale topographies also could have a major physical containment of microscale contact guidance, modulating the cell response, and inducing changes in adhesion, gene expression, and differentiation [19,20,21,22,23,24,25,26,27,28]. Control of cell fate, especially of stem cells by modulation of cell spatial orientation and spreading, would provide an increased impact for tissue engineering and regenerative medicine applications [27,28,29]. By reprogramming cells toward needed phenotypes, specific therapeutic strategies could be designed to replace or repair damaged tissues.

Nanotopographical approach alone, by exposure to controlled semi-random nano cues, was demonstrated to induce higher levels of mesenchymal stem cell (MSC) osteoinduction [30,31,32] than on specific highly ordered (both produced by electron beam lithography, EBL) or random nanostructures (as produced by e.g., anodization, milling polishing, sandblasting, etching, and blasting) [32]. Nevertheless, the main disadvantages relate to surface contamination, chemical changes, and the lack of control over the reproducibility of the surface’s topography.

Femtosecond (fs) laser surface modification is used as a debris- or contaminant-free technique that can confer automatized, reproducible, fast time processed surfaces with increased roughness and stable characteristics. Due to low thermal loading and the ability to maintain the bulk properties of the material after fs laser processing, the disadvantages of other previously described surface modification techniques could be overcome [33], and high-quality microstructures and machining precision could be obtained as an exciting alternative to those obtained by conventional surface treatments of Zirconia surfaces [34,35,36,37,38,39,40].

Besides the topographical and chemical characteristics of the Zirconia biointerfaces, another issue is represented by the lack of knowledge of a more realistic model for in vitro biotesting. Until now, the in vitro studies on the biological response to Zirconia entailed mainly investigating the activity of osteoblast and osteoblast-like cells, fibroblasts, and epithelial cell lines [41,42]. There are few studies related to the interaction of MSCs with micro-nanotopographies generated in Zirconia-based materials, treating mainly pit-like or ditch-like topographies. Our previous results, for example, show that droplet-shaped microcavity arrays lead to an increased surface area inducing cell morphology modification toward a polygonal shape and, consequently, increases the circularity of the stem cell nuclei [35]. The cytoskeleton filaments extend jointly over their combined granular and ripple-like structures present within each cavity [35]. These preliminary findings of both circular micropatterns and the presence of nano ripples influencing MSCs were confirmed recently by Stanciuc et al. [39] by using pit arrays with diameters from 10 to 30 µm and two different structure depths. The pattern, built as a 30 µm diameter pit/10 μm depth, was proposed to induce the most substantial osteoblastic human mesenchymal stem cell (hMSC) commitment based on morphological parameters shaping cell commitment, compared to smaller pattern features. Still, the evaluation of the osteogenic commitment of hMSCs over long-term cell cultures has yet to be reported in the literature. Other preliminary data on ditch-like structures obtained by fs laser ablation on pre-sintered polished Alumina-Toughened Zirconia ATZ disks, using a Yb:KYW chirped-pulse-regenerative amplification laser system (1030 nm, 500 fs) show the alignment of hMSCs on the developed microgroove surfaces (width < 20 µm with the depth of approximately 2.6 µm), as well as more efficient osteogenic differentiation onto the micro-grooved surfaces [43,44].

Nevertheless, there is still an increasing need for designing Zirconia structures to tailor the osteogenic response and study more in-depth the influence of broader types of topographies for influencing the osteoblast differentiation. Considering recent reports on the mixed micro/nanoscale approach to mimicking features similar to osteoclast-bared resorption pits/trenches enhancing an osteogenic response [45], and the known fact that controlled geometric shapes trigger a specific cellular response [46], we propose in our study isotropic- and anisotropic-defined geometric microshapes with nanoroughed sidewalls as surfaces designed for stimulating an osteogenic response.

Therefore, we obtain, using direct fs laser texturing, new anisotropic and isotropic topographies (microgrooves delimited by rigid or curved ridges, and overlapped crossings of the former arrays, respectively), by maintaining the distance edge-to-edge within the size range created by osteoclasts during their bone remodeling activity. Bone progenitor cells (hMSCs) are used to assess the potential of different Zirconia micro-nanotopographies to support their adhesion, proliferation, and osteogenic activity as compared to a non-processed, flat material.

The designed structures, consisting of a combination of micron-sized structures and nanoroughed walls, are used as contact guidance elements for influencing the cytoskeletal organization and developing bioinstructive topographies that can then be translated to implant materials designed for specific bone tissue regeneration, such as BAHA abutments.

## 2. Materials and Methods

### 2.1. Surface Patterning of Isotropic and Anisotropic Microtopographies

**Figure 1:**
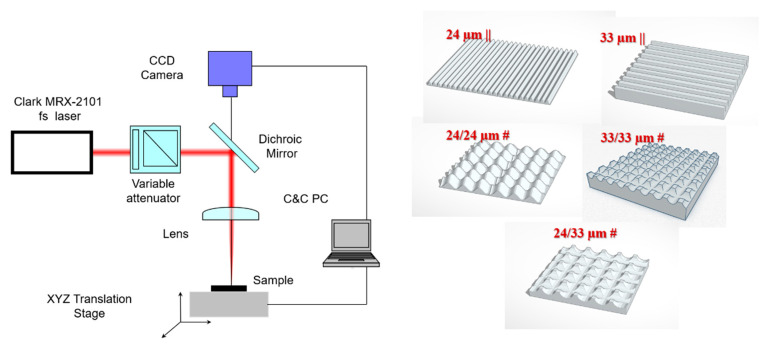
Experimental set-up, the Zirconia control, and the proposed textured designs.

The material under investigation was yttria, partially stabilized Zirconia sheet, cut with the dimension of 10 × 10 × 5 mm^3^ from Zirkonzahn (Zirkon Translucent-ZRAB0490, Lot ZB 0070A, Norcross, GA, USA). It was used as received prior to the laser irradiation. A Ti: Sapphire regenerative amplifier laser (Clark MRX-2101, Dexter, MI, USA), emitting a wavelength of 775 nm, with duration pulses of 250–300 fs, and a 2kHz repetition rate was used in this study. The experimental set-up was described previously [46]. Briefly, the samples were placed on a motorized platform with three-axis motion, x, y, and z. The focusing element used was a 75 mm convex lens which gave an estimated focused spot area of 0.6 mm^2^ on the Zirconia samples positioned on the XYZ computer-controlled translation stage system. A series of experiments were conducted for 2 J cm^−2^ fluences, while the traverse speed was set at 0.2 mm/sec (Figure 1).

A high-sensitivity thermal sensor type 3AP-Ophir (Jerusalem, Israel) was placed in front of the attenuator and was used for laser energy measurements. Laser pulses were attenuated using a variable attenuator composed of a half wave-plate and a Glan-laser polarizer. A monochrome CCD camera connected to a PC was used for monitoring the irradiation process in real-time. During each irradiation, the laser beam was focused directly onto the sample. We aimed to maintain the distance between the ranges of the bone resorption trench widths. Therefore, the sample areas surfaces were fully textured by its translating to the laser beam with steps of 24 µm or 33 µm, either only in parallel lines leading to specific anisotropic patterns (such as parallel grooves/lines hereafter named 24 µm || and 33 µm || obtained by translating steps of 24 µm and 33 µm, respectively), or in perpendicularly overlapping the two chosen translation steps, leading to isotropic superimposed crossline microgroove arrays or pillar-like arrays (hereafter named 24/24 µm #, 33/33 µm # and 24/33 µm #). The chosen steps aimed to obtain curved (by 10% beam overlapping) and respectively stiff-edged surfaces, as well as maintain the distance between structures (edge-to-edge, or in the case of wavy ones, from center-to-center) similar to those found and measured within bone resorption trenches (in the range of 22–35 µm) (Figure S1). Control flat Zirconia substrates were used as a comparison throughout our study.

### 2.2. Scanning Electronic Microscopy (SEM)

Morphological observation of the structured surfaces was undertaken by means of a JSM-531 Inspect S scanning electron microscope (SEM), (Hillsboro, OR, USA) at accelerating voltages between 5 and 20 kV. The samples’ chemical evaluation was performed using energy dispersive X-ray spectroscopy with 10 kV accelerating voltage (EDAX, Element 2CB detector on a JSM-531 Inspect S Electron Scanning Microscope, FEI Company, Hillsboro, OR, USA). Samples were coated with 10 nm Au before imaging to obtain electrical conductivity. Regarding the case of SEM analysis of cell-Zirconia interfaces, after three washes in phosphate buffer saline (PBS), human mesenchymal stem cells (hMSCs) were dehydrated by sequential immersion in 70, 90, and 100% ethanol, 15 min twice for each concentration. Samples were air-dried and metalized by using a sputtering coater (Agar Scientific Ltd., Essex, UK) with 10 nm Au prior to microscopy investigations.

### 2.3. Atomic Force Microscopy (AFM) and Profilometry Measurements

Atomic force microscopy (AFM) (XE 100 AFM setup from Park, Suwon, Korea) measurements in the non-contact mode were performed to analyze the obtained features in several different areas and dimensions.

The surfaces of the samples also were evaluated using a profilometer at a scanning rate of 100 µm/s and an applied mass of 0.5 mg (KLA Tencor P-7 contact profilometer, Milpitas, CA, USA). APEX 3D BASIC V7 dedicated software (Milpitas, CA, USA) was used to determine the height profiles created by laser texturing and to evaluate overall roughness values.

### 2.4. Contact Angle and Surface Energy Measurements

The hydrophilic or hydrophobic states of Zirconia surfaces were evaluated by measuring the contact angle of 4 µL deionized water placed on the substratum, 3 s after drop positioning. The contact angle measurements were performed in static mode using an optical measuring system (CAM101, KSV, Finlanda-Biolin Surface, Espoo, Finland) provisioned with a video camera with a FireWire interface allowing the acquisition of images with a resolution of 640 × 480 pixels. The sessile drop method was applied at constant room temperature (20 °C). The contact angle’s reported values were obtained upon averaging 3 measurements performed on different areas of the sample.

Surface free energy (SFE) was measured using the contact angle measurement of two wetting agents: water, di-iodomethane. This calculation was conducted using the concept of polar and dispersion components using the Owens, Wendt, Rabel and Kaelble (OWRK) method for estimation [47,48,49].

### 2.5. Sterilization Procedure

All Zirconia samples were sterilized before cell culturing by autoclaving in water vapors at 121 °C at a pressure of 1 atmosphere for 30 min in a Falcon 30 Autoclave (LTE Scientific, Greenbridge Lane, Greenfield Oldham, OL3 7EN, UK).

### 2.6. Cell Culture

Human mesenchymal stem cells (hMSCs conform with Ethics Committee approval Univ. Med. Pharm., Craiova, Romania (ref. No.68/11.07.2016). Biosecurity Commission approval, IBAR (ref. No. CBS19_06/3.10.2019)) were isolated on Ficoll™ gradient from bone marrow aspirates, as previously described [50]. Cells were then cultured in Dulbecco’s Modified Eagle’s Medium (DMEM) supplemented with 10% fetal calf serum (FCS), L-Glutamine (L-Gln), and Penicillin/Streptomycin (Pen/Strep) (all from Gibco, Thermo Fisher Sci., Waltham, MA, USA). They were allowed to grow at least two passages before use in experiments. Cells were seeded onto Zirconia samples at a density of 5000 cells/cm^2^ in 24-well or 48-well plates (Sarstedt, Corning) and cultured for the specified periods.

### 2.7. Osteogenic Differentiation

Regarding in vitro osteoinduction assays, human mesenchymal stem cells (hMSCs) were cultured in differentiating conditions using an α-MEM complete medium (Gibco, Thermo Fisher Sci., Waltham, MA, USA) supplemented with 82 μg/mL of ascorbic acid (Santa Cruz, Dallas, TX, USA), 100 nM dexamethasone (Sigma-Aldrich, Saint Louis, MO, USA), and 10 mM β-glycerophosphate (Calbiochem, EMD Chemicals, San Diego, CA, USA). The medium was changed twice per week up to 28 days of culture.

### 2.8. Microscopy Sample Processing

Occurring at 3 h or 3 days post-seeding, human mesenchymal stem cells (hMSCs) were fixed at room temperature (RT) using a solution consisting of either 4% para-formaldehyde (for immunofluorescence analysis, 20 min) or 0.25% glutaraldehyde (for scanning electron microscopy (SEM), 45 min).

#### 2.8.1. Immunofluorescence

After three washes in PBS, cells were permeabilized using a 0.2% Triton-X-100 (Sigma, Saint Louis, MO, USA) for 3 min at RT and then washed again with PBS. Anti-vinculin (1:150, #V9264, Sigma, Saint Louis, MO, USA), anti-zyxin (1:100, Invitrogen, #396000, Thermo Fisher Sci., Waltham, MA, USA), anti-Ki-67 (1:100, #MA5-14520, Thermo Fisher Sci., Waltham, MA, USA), or anti-osteocalcin (1:100, #AB10911, Chemicon, Temecula, CA, USA) primary antibodies were used to stain focal adhesions, proliferating nuclei, or differentiated cells, respectively, all diluted in 0.5% BSA-PBS. Secondary Alexa Fluor 594 antibodies (1:400) were used next, while actin filaments were labeled using Alexa Fluor 488-conjugated Phalloidin (Life Technologies, Thermo Fisher Sci., Waltham, MA, USA) for 30 min at RT in the dark. Excess staining solution was removed by PBS washing and specimens were mounted using Vectashield with 4′,6-diamidino-2-phenylindole DAPI (Vector Laboratories, Burlingame, CA, USA) and by overlaying a rectangular cover slip (Marienfeld, Lauda-Königshofen, Germany).

#### 2.8.2. Confocal Microscope Imaging

Samples were imaged using a Carl Zeiss LSM-710 confocal microscope (Jena, Germany). Fluorescent 2D z-stacked images were obtained using Zen software (Carl Zeiss Microscopy, Jena, Germany) and the 63×, 1.4 NA objective. Orthogonal projections (frontal xy, transverse xz, and sagittal yz) and 3D reconstructed images were generated using Image J (1.52v) to visualize cell distributions on different topographies.

#### 2.8.3. Quantitative Image Analysis

Regarding image cytometry analyses, whole samples were automatically scanned, and images were captured with a 20× objective using a TissueFAXSiPlus imaging system controlled by the TissueFAXS software module (TissueGnostics, Vienna, Austria).

Based on DAPI fluorescence, morphological parameters of nuclei were quantified. Using the TissueQuest software (TissueGnostics, Vienna, Austria), we determined the area and Feret ratio parameters. The Feret ratio represents the ratio of the minor axis divided by the major axis of the nucleus, thereby defining its circularity index. The relative frequency of nuclei in each area and the circularity index category were represented as violin plots. Up to a total of 100 nuclei were analyzed from the two replicates of each condition to determine the percentage of proliferation marker protein Ki-67^+^ human mesenchymal stem cells (hMSCs) [51], nuclei size and elongation.

Regarding quantification of cell shape parameters (cell area, perimeters, orientation angle, Feret ratio), image analysis was performed using ImageJ software from the National Institute of Health (Bethesda, MD, USA) (free download available at http://rsb.info.nih.gov/ij/) on grayscale representations of each field of view. Based on fluorescent staining of actin filaments, and using automatic thresholding, cell morphological parameters were quantified. Cells placed at the image boundary or found in contact with other cells were excluded from the analysis, as described before [52]. Using automated detection of cell outlines, areas and perimeters of adhered cells were determined. Cell lengths and widths were calculated by fitting the object to an ellipse and software calculation of major and minor axes. The angular orientation of each cell was calculated as well. To investigate cell alignment to laser-ablated structures, the deviation angles between cells and ridges/grooves were calculated as the absolute differences between the orientation angles of lines (or interrupted lines) and the orientation angles of the cells’ long axes. Concerning planar control, the orientation angles were corrected by 180° for cells found at more than 90°. A minimum of 100 cells were analyzed from the two replicates of each condition.

Cells and nuclei were considered aligned if the angle between their major axis and the micrograting was <15 °C.

#### 2.8.4. Cells Analysis by Scanning Electron Microscopy (SEM)

After fixation, samples were washed two times with PBS and dehydrated by successive immersion in 70, 90 and 100% ethanol (EtOH), twice for 15 min for each concentration. Then, subsequent incubations in 50%: 50%, 25%: 75% and 0%: 100% solutions of EtOH: hexamethyldisilazane (HMDS) twice for 3 min for each treatment were performed. Samples were left out to dry inside the fume hood and then were covered with a 10 nm thin gold layer deposited prior to analysis.

### 2.9. Alizarin Red Staining

Regarding detection and quantification of mineralization at 28 days after culturing human mesenchymal stem cells (hMSCs) in osteogenic conditions on the proposed surfaces, we employed Alizarin Red staining of matrix accumulated calcium. Cells were gently washed using PBS and fixed for 20 min with 4% Paraformaldehyde (PFA). After washing with distilled water, samples were incubated with Alizarin Red S solution (40 mM, pH 4.1–4.3) at room temperature for 30 min. The unbound excess dye was removed by washing twice with water. Samples were photographed using a smartphone. Concerning quantification, the dye was extracted for 15 min at room temperature using a 20% methanol and 10% acetic acid solution in water. The supernatant was transferred to 96-well plates and the absorbance read at 405 nm using a Mithras LB940 spectrophotometer (Berthold).

### 2.10. Statistical Analysis

All statistical analyses were performed using GraphPad Prism version 8.2.0 for Mac, GraphPad Software, San Diego, CA, USA, www.graphpad.com. An unpaired two-tailed Student’s *t* test was used to compare the statistical significance of differences in area, perimeter, and elongation values for cells and nuclei for each proposed structure compared to the unprocessed flat control. (differences: * *p* < 0.05, ** *p* < 0.01). Since the data on orientation angles were not normally distributed, they were analyzed using the Kruskal-Wallis one-way analysis of variance followed by Dunn’s multiple comparison test (differences: * *p* < 0.05, ** *p* < 0.01, *** *p* < 0.001).

## 3. Results and Discussions

### 3.1. Design of Structure Arrangements Textured in Zirconia Ceramic Substrate and Surface Characterization

When designing a bioinstructive mechanical microenvironment benefic to progenitor cell osteogenic commitments, an enhanced cytoskeleton stretching is essential [53,54,55,56,57]. Given the fact that Zirconia does not naturally form a direct bond with bone [58,59], improving its surface properties by laser texturing, and understanding cell behaviour to increase their pro-osteogenic properties, still represents a challenge. Moreover, it is known that cells behave differently on rectilinear versus curved surfaces, and suppression of cell adhesion and proliferation onto the concave microscaled structures was observed due to cell plasma membrane deformation and subsequent opening of membrane channels onto curved concave structures [60,61]. Within this context, our design entailed isotropic structures: i.e., micropillars with a curved top surface or rectangular micrometric flat tops, as well as their equivalent superimposed microridges/grooves anisotropic arrays.

**Figure 2:**
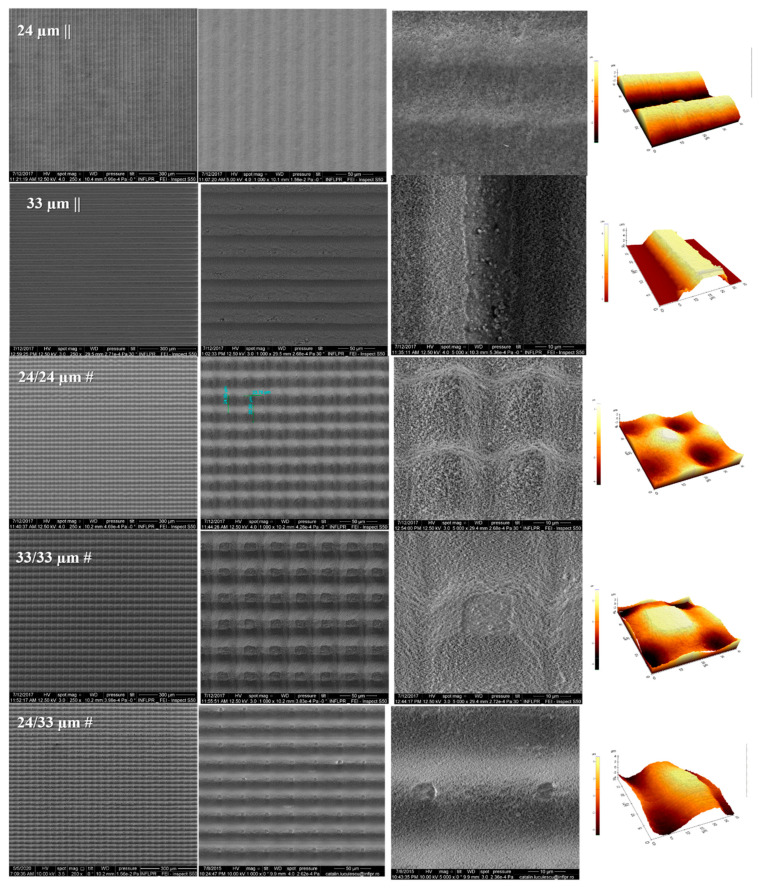
Scanning electron microscopy (SEM) and atomic force microscopy (AFM) images of isotropic (24 µm || and 33 µm ||) and anisotropic textured surfaces structures (24/24 µm#, 33/33 µm #, and 24/33 µm #).

Thus, anisotropic arrays of lines/grooves were obtained, with the ridge top width of approximately 0.9 µm (24 µm step—wavy profile—attenuating the abrupt profile characteristics of grooves and ridges and providing a surface curvature for cell surface interaction) and a 10 µm ridge top width, respectively (for the 33 µm step), as shown in Figure 2 and Table S1.

When samples were translated in both the XY directions with a 33 µm step (33/33 µm #), pillars with a square top (side of 10 µm) were developed, while a cross-step of 24 µm (24/24 µm #) led to a pillar with a 0.9 µm top width (slightly curved). Alternating the two steps in the xy direction (24/33 µm #) resulted in rectangular top pillars (sides of 5 µm) ablated in a Zirconia substrate (Figure 2).

Flat top structures were characterized by an approximately 4.5 µm depth, while the wave-like structures were characterized by depths of 3.5 µm (Figure S1). The double-crossing of the laser beam for creating grids/pillar-like structures led to a maximum depth of 8.2 µm between the highest and lowest sites at the intersection points, as measured by atomic force microscopy (AFM) (Figure S1). The standard deviations were maintained below 1 µm (Table S1, Supplementary Materials).

Moreover, the resulting pillar-like structures resulting from crossing the lines and the height of these structures (~3.5–4.5 µm) were designed as a hypothesis that the multiscaled surfaces could stimulate the membrane tension of the cells as a result of the adherence onto it, with a beneficial effect on the osseogenic response. The structures corresponding to 33 µm || were characterized by stiff edges and increased roughness of the walls compared with the flat non-processed one (Figure 2). A significant change from stiff edges to curved ones was created by a 10% crossover of the laser beam on the irradiation area, an attenuation of the abrupt profile characteristic to grooves and ridges being obtained, thus providing a surface curvature for cell surface interaction.

Shown in Table S1, taken from the profilometry measurements, the roughness depending on both profile and 3D areas increased significantly after the laser texturing process, but no significant differences were observed between the measured roughness within the sidewalls of the structures or at the bottom of the structures, not resembling the previously ripple-like structures observed in circular patterns by Dinca et al. [35] and Stamatiuc et al. [39].

**Figure 3:**
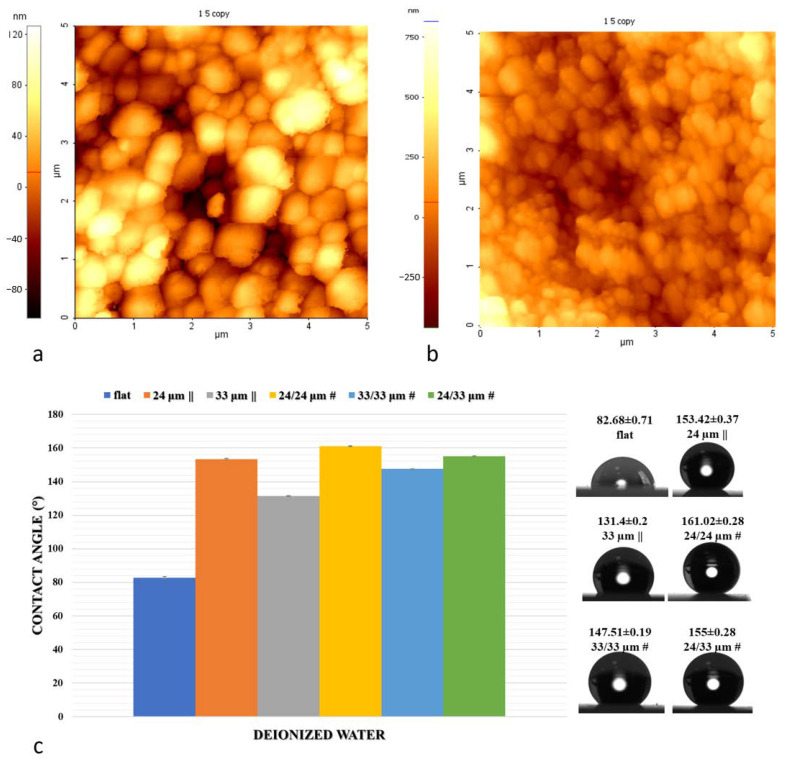
Atomic force microscopy AFM images of typical surfaces of the samples before (**a**) and after irradiation (**b**). (**c**) Contact angle measurements.

All irradiated substrates exhibited granular structures onto the walls and bottom. When the grain sizes for the control samples were in the 300–400 nm range, there was a decrease in grain size to 180–250 nm for the textured sidewalls. (Figure 3).

Nanoscale features, however, are orders of magnitude smaller than most mammalian cells, including osteoblasts. The features are, in fact, at the same scale as filopodia, which are actin-driven membrane projections (50–100 nm diameter tips) that cells use to probe the surface. Therefore, when considering only a limited area of 5 µm × 5 µm, as for cellular contact surfaces, a roughness change for the inner surfaces of the structured regions compared with the control group was noticed. Within this context, when compared with the untreated flat sample (Ra of 91 nm), the femtosecond (fs) laser treatment brought about a consistent surface modification on the Zirconia sample grain sizes within the groove’s walls (Ra of 182 nm) (Figure 3).

Previous studies of the authors have shown that as-sintered Zirconia surfaces (without sandblasting) have a small grain size of ~0.38 µm and a roughness (Ra) of ~0.43 µm [62]. Nevertheless, in the present study, the medium roughness was below the reported values (123 nm). To contrast, the roughness corresponding to textured areas surpassed the reported level of microroughness (<1 µm) that was shown to favour the cell growth and attachment [63]. Here, for the anisotropic patterns, the medium value was maintained below 2 µm in contrast to the isotropic pillar arrays, where roughness in all cases was above 2 µm (Table S1).

### 3.2. Wettability and Surface Free Energy Measurements

The changes in Zirconia surface topography at the micro- and nano-scales have impacted properties such as wettability and surface free energy and, consequently, will affect how cells interact with a surface. The surface wettability of all structured surfaces had highly significant differences compared to the control surface, reflecting a strong hydrophobicity increase with texturing. It is shown that the surface becomes more hydrophobic with increasing surface roughness (Figure 3c). Regarding the case of 24 µm || and 24/24 µm #, when the features on the rough surface have relatively smoother aspect ratios (curved areas as opposed to sharp edges), droplets do not thoroughly wet the surface (corresponding to the Wenzel model) (Figure 3c).

Nevertheless, the presence of sharp edges and spaces between the grooves led to a less than 20% decrease in contact angle values, depending on the droplet being measured perpendicularly or oblique to the samples.

Beside the impact on the contact angle of the surface, the changes in surface topography at the macro-, micro- and nano-scales are known to impact properties such as surface free energy, which will consequently affect cell interactions with the surface. Despite a few reports on how ceramics, in general, are classified as having ‘high energy’ surfaces being supportive of cell attachment and spreading, there are very few studies showing the clear effects of changes in the surface free energy of ceramics on cell behavior (as fundamentally altering their surface free energy is difficult). Here, texturing Zirconia samples was shown to increase the surface free energy (γ) values compared to the flat control by augmenting surface area. The initial SFE value of the control surface (31.52 mN/m) (Table 1) is much lower than those of the textured surfaces (values up to 65.18 mN/m). M. L. Gonzalez-Martin et al. [64] reported a 42.61 SFE value for ZrO_2_, stating that the acid-base component probably depends on the density of -OH groups on the surface of the solids studied.

The values of γ ranged between 31.52 and 65.18 mN/m; and the highest γ was determined for 24/24 µm # (65.18 mN/m) and 33/33 µm # (63.38 mN/m). Significant differences were observed for the polar surface value (γ p), which is considered an essential factor affecting biological interactions. Hao and Lawrence [65] observed that the thickness of the adsorbed human serum albumin decreased as the polar surface energy of the magnesia partially stabilized zirconia increased. While no significant differences were observed for the disperse component γ d, the polar component γ p was determined as the highest for the isotropic topographies (24/24 µm #, followed by 33/33 µm # and 24/33 µm #), with lower values for anisotropic topographies (24 µm || and 33 µm ||) (Table 1, indicating that the surface free energy will have a predominantly non-polar nature, given by the secondary (van der Waals)-type bonds.

### 3.3. Human Mesenchymal Stem Cell Adhesion and Guidance onto Microtextured Zirconia

**Figure 4:**
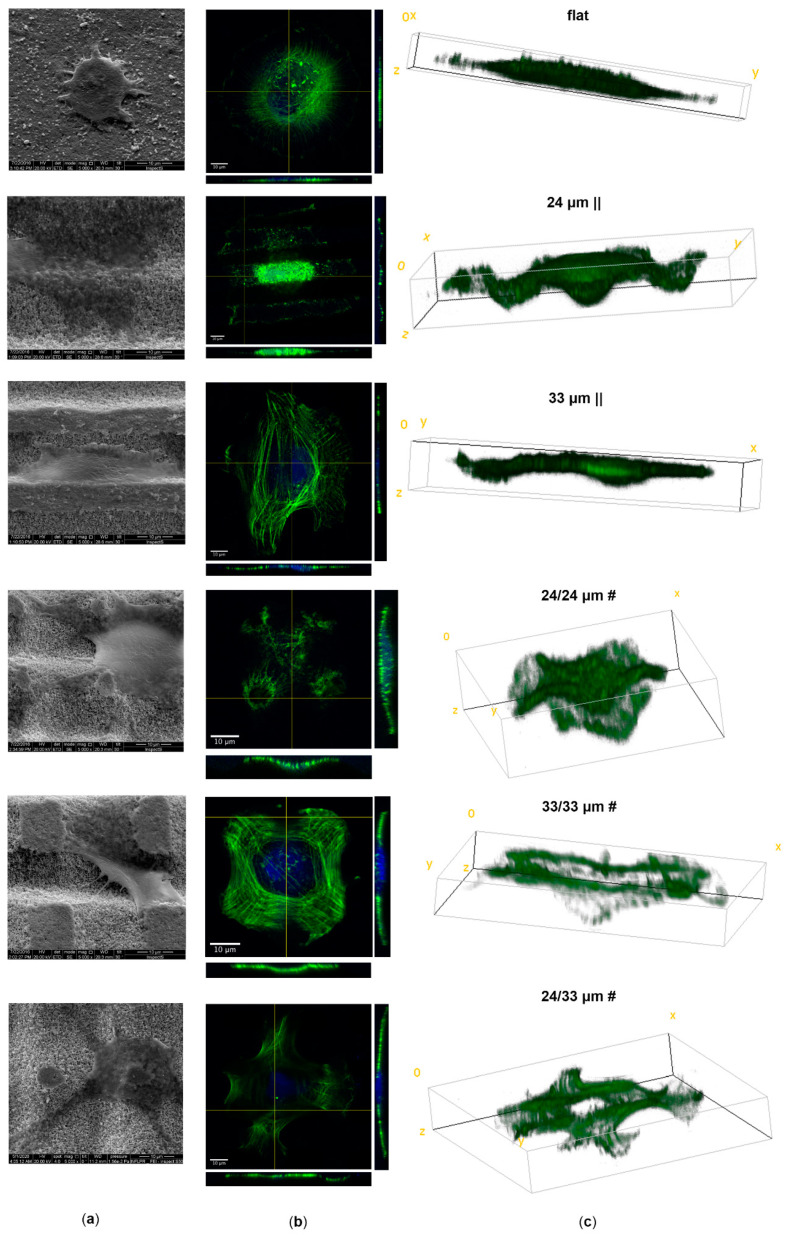
Early attachment of human mesenchymal stromal/stem cells (hMSCs) onto Zirconia microtopographies. Scanning electron microscopy (SEM) (**a**), orthogonal sliced confocal microscopy images (**b**) (scale bars = 10 µm), and their corresponding 3D rendering (**c**) at 3 h post-seeding onto the tested biomaterials. High-magnification SEM images are presenting individual cell interactions with each of the structured Zirconia substratum. The fluorescence images were obtained upon staining of actin filaments with Alexa Fluor 488-Phalloidin (green) and nuclei with Hoechst (blue). Image acquisition was performed using the Zeiss LSM-710 laser scanning microscope.

Given that a bone-anchored hearing aid (BAHA) bone-implant success relies on the optimal embedding of the prosthetic piece into the skull of the patient presenting with hearing defects, we next investigated the interaction of laser-textured Zirconia substrates with human mesenchymal stromal/stem cells (hMSCs), which are osteoblast precursor cells. Bone progenitor cell attachment, proliferation, and new bone tissue formation at the temporal bone-implant interface are vital steps in obtaining implant osseointegration. We first assessed early hMSC attachment onto the proposed anisotropic and isotropic Zirconia patterns using scanning electron microscopy (SEM) and fluorescence microscopy (Figure 4 and Figure S2). Occurring 3 h after cell seeding, we observed specific changes in cell shape function of the material topography using SEM (Figure 4a), as well as upon confocal analysis of actin filament staining (Figure 4b,c). Cells grown on the control’s unprocessed flat surface showed a predominant round shape (Figure 4a,b; Figure S2a,b), and limited spreading, with the thickest cytoplasm present around the cell nucleus (Figure 4c). When cells were seeded onto the anisotropic line patterns they adopted a more elongated morphology, with the more rigid structure (33 µm ||) inducing a more enhanced stretching, compared to the wavy/curved (24 µm ||) surface. Cells intimately follow material topography in both cases (Figure 4a–c), with the nucleus being almost entirely deposited in grooves (autofluorescent stripes in Figure S2a), while being occasionally exposed on the top curvatures of the 24 µm || processed Zirconia (Figure 4b) also. This is consistent with reports from Karine Anselme’s lab [54]. Atop the isotropic patterns, cells attach mainly to the pillar edges, while the nuclei decant between pillars (Figure 4a–c); Figure S2a). The orthogonal (xz,yz) slicing (Figure 4b), as well as the 3D rendering (Figure 4c), provide evidence that hMSCs follow near the 24 µm || and 24/24 µm # microtopographies. To compare, they adopt a shallower appearance on structures obtained using the 33 µm irradiation step. This could be explained by the more rigid exposed structures and increased depth of the latter, which apply more stress on the cell cytoskeleton [66]. Interestingly, on the 33/33 µm # structures, most nuclei are displaced laterally from the cell center (Figure S2a,b); this emphasizes the impact of the substrate topography of these specific isotropic patterns, which impose the highest stretch on the cytoskeleton at the groove intercrosses, where depth can reach up to ~8.5 µm.

**Figure 5:**
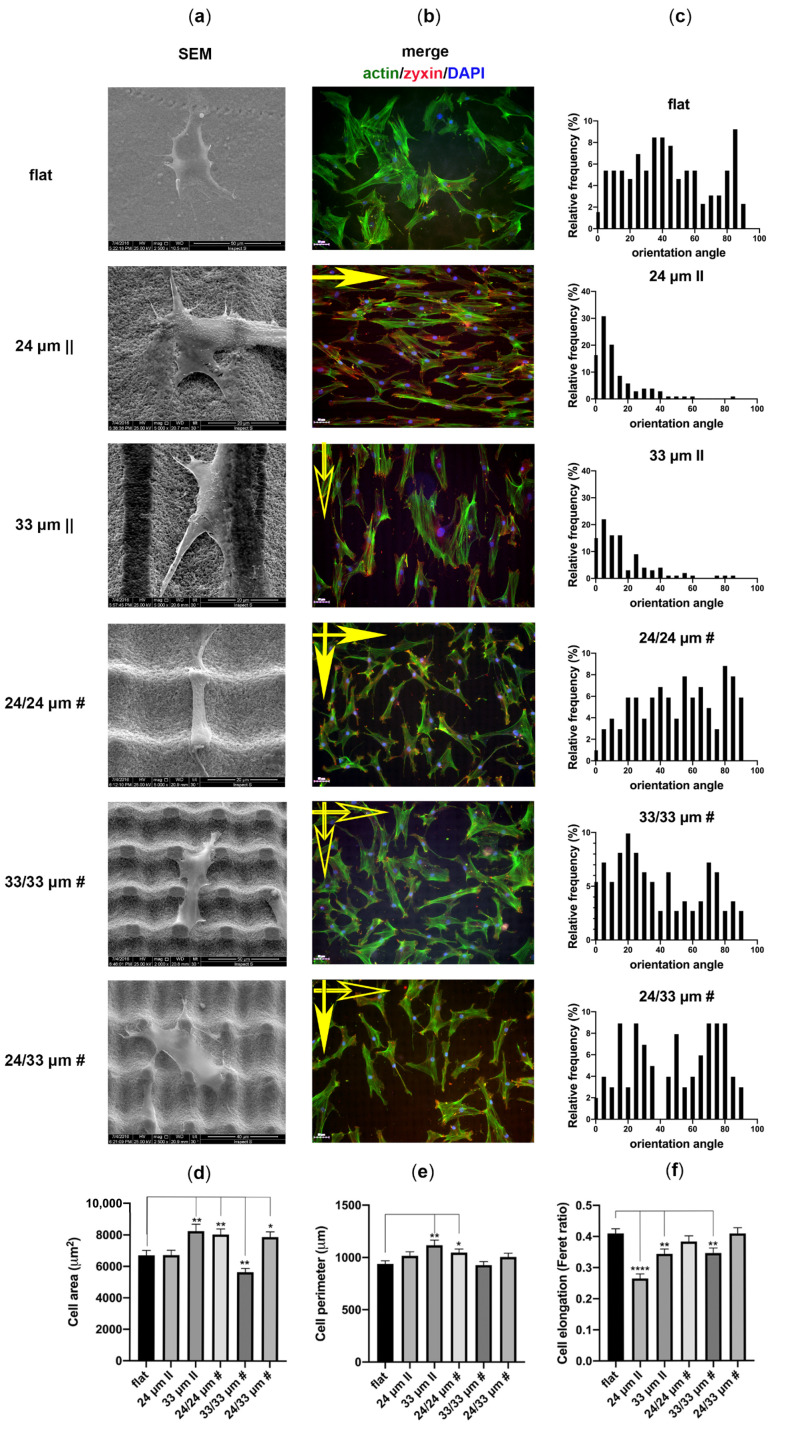
Adhesion of human mesenchymal stromal/stem cells hMSCs onto Zirconia microtopographies. A. Scanning electron microscopy (SEM) (**a**) and immunofluorescence microscopy (**b**) images, and their corresponding quantifications (**c**–**f**) at 3 days post-seeding onto the tested biomaterials. High-magnification SEM images (**a**) are presenting individual cell interactions with each of the structured Zirconia substratum. The fluorescence images (**b**) were obtained upon labeling with antibodies for zyxin (red), staining of actin filaments with Alexa Fluor 488-Phalloidin (green), and nuclei with Hoechst (blue). Arrows indicate the direction of irradiated line structures (full arrow for 24 µm step; empty arrow for 33 µm step). Percentage of cells for each orientation angle versus the horizontal direction are shown in frequency distribution histograms (**c**). Quantitative measurements of cell areas (**d**), perimeters (**e**) and elongation (**f**) are shown in graphs. Image acquisition was performed using the TissueFAXS iPlus automatic system. Data represent mean ± S.E.M., * *p* < 0.05, ** *p* < 0.01, **** *p* < 0.0001.

**Figure 6:**
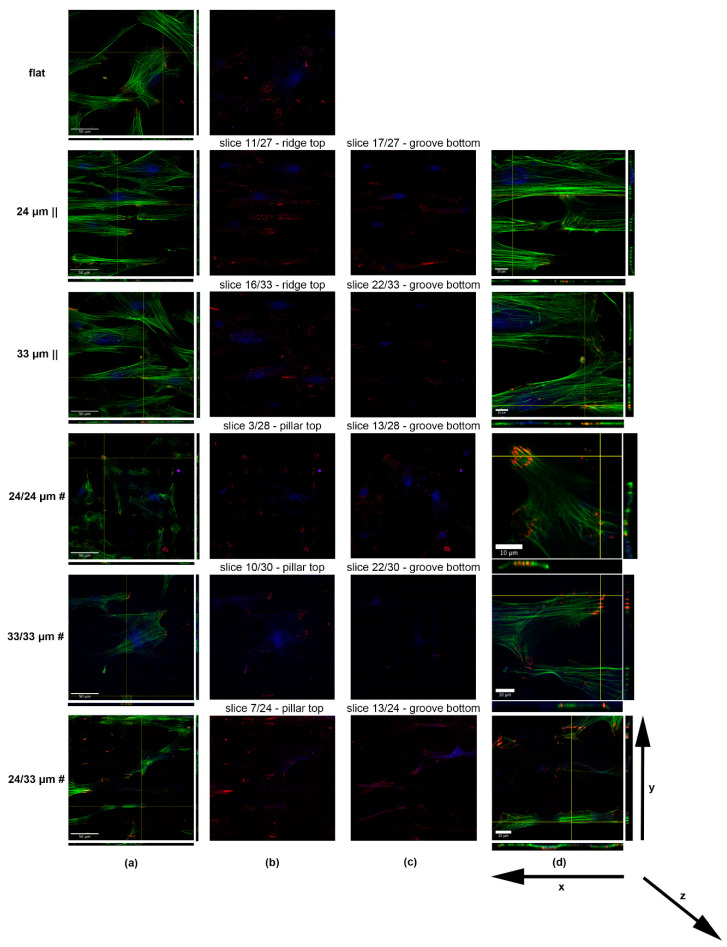
Confocal images (**a**) and orthogonal slices (**b**,**c**) depicting focal contacts of human mesenchymal stromal/stem cells hMSCs with the top (**b**) and bottom (**c**) structures of the Zirconia microtopographies. Representative cells are shown to depict the zyxin-labeled focal adhesions. Detailed views of single cells and orthogonal slicing thereof are provided in (**d**). Image acquisition was performed using the Zeiss LSM-710 laser scanning microscope.

Next, we analyzed hMSC morphology, adhesion, spreading and orientation at 3 days after seeding onto Zirconia microtextured surfaces (Figure 5; Figure 6). The SEM and fluorescence microscopy images reveal that hMSCs adopt an elongated morphology parallel to the microgrooves while growing on the anisotropic patterns, as proof of their contact guidance action (Figure 5a,b). When adhered to the isotropic grid-like patterns, cells spread more freely and orient their bodies guided by the bi-directional landscape (Figure 5a,b). We quantified the cell orientation angles on all proposed Zirconia structures and their areas and perimeters (Figure 5c).

Results show that a large proportion of the cells orient their bodies at angles less than 20 degrees when grown on the 24 µm || topography and (histograms of angle frequency distribution in Figure 5c) at angles less than 30° on the 33 µm || topography. This shows that the 24 µm || processed Zirconia induces higher contact guidance than the looser 33 µm || surface. Concerning the isotropic 24/24 µm # substrate, cells adopt a semi-random orientation, similar to the flat control. Interestingly, cells grown on 33/33 µm # tend to align at approximately 20 degrees to both cartesian guides (~20 and ~70 degrees relative to the horizontal reference in the image, Figure 5b), while those grown on the hybrid 24/33 µm # pattern tend to follow the guidance of the 24 µm || direction, which becomes dominant (~70–80 degrees relative to the horizontal reference in the image, which is the 33 µm || direction, symbolized by the empty arrow in Figure 5b). A smaller peak in the 24/33 µm # angle distribution histogram depicts cells that align at ~20 degrees to the 33 µm || direction, which results in a secondary determinant of cell orientation. Cell response to guiding cues determined modifications in spreading (as revealed by area and perimeter quantifications, Figure 5d,e) and in elongation (as determined based on the Feret ratio, Figure 5f). An increased cell spreading was observed on the 33 µm || topography (Figure 5d,e; Figure 5b), while hMSCs grown on the 24 µm || did not show significant changes compared to the flat control. Considering the isotropic patterns group, the 24/24 µm # and 24/33 µm # topographies imposed and increased cell spreading, while the opposite was observed for the 33/33 µm # structure (Figure 5d,e; Figure 5b). The topographies that increased hMSC elongation were the anisotropic 24 µm || pattern, followed by the 33 µm || and, interestingly, by the isotropic 33/33 µm # structure (Figure 5f).

Overall, these results indicate that the 24 µm || processing forces hMSC elongation due to topography restrictions, with virtually no impact on cell area and perimeter (Figure 5d–f). The 33 µm || pattern produces milder elongation and increases spreading, thereby allowing neighboring cells to interact (Figure 5d–f; Figure 5b). The isotropic 24/24 µm # and 24/33 µm # topographies increase spreading compared to the flat control, with no significant effect on elongation (Figure 5d–f). Interestingly, the 33/33 µm # structure decreases cell area, while producing elongation (Figure 5d–f), mostly by restricting cell adhesion between created pillars (Figure 5a), which generates alignment along both cartesian directions (Figure 5b,c).

To analyze cell anchoring points relative to surface microtopography, we sought to test zyxin expression at focal adhesion sites, consistent with its described role as an actin cytoskeleton stabilizer, sensitive to mechanical tension [67]. Its recruitment to focal adhesions is induced by cell stretching [68,69]. The zyxin positive focal adhesions (FAs) are distributed at the filopodia tips, where they are contacting the substrate (Figure 5b, Figure 6a–d, red), thereby anchoring the actin cytoskeleton (Figure 5b, Figure 6a–d, green) to the focal adhesion sites, where the filaments colocalize with zyxin (Figure 5b, Figure 6a–d, yellow). Regarding the cells on the unprocessed Zirconia, we observed a predominant polar distribution of zyxin FAs at the cell front and rear edges (Figure 6a,b). Concerning the 24 µm || processed substrate, cells were anchored to the material mainly by contacting the ridge sides (Figure 6b) with only a few adhesions forming within the grooves (Figure 6c). This led to a parallel organization of actin filaments to the ridge/groove pattern (Figure 6a,d). The orthogonal slicing showed that cells are attached to the upper exposed ridges, while the nucleus sits at the bottom of the groove (Figure 6d-yz). The 33 µm || ridges/grooves pattern is more widely interspersed than the 24 µm || structure. Hence, the density of FAs per cell overlapping ridges is decreased and the cell curvatures on the 33 µm || are more shallow (Figure 6a,b,d-yz). Here, only a few FAs are present within grooves (Figure 6c). When exposed to the 24/24 µm # processed surface, cells anchor their actin cytoskeleton by forming rings of FAs surrounding the microstructure peaks (Figure 6a,b,d-xz). Fewer contact sites are detectable within the intercrossed regions (Figure 6c). The cells adhere almost entirely to the rectangular inter-grid spaces when grown onto 33/33 µm # Zirconia (Figure 6a,b,d-xz,yz), most probably due to difficulty in contacting the deep ~8.5 µm laser-processed material.

As a consequence of the increased stretching, the zyxin staining revealed elongated mature FAs at the cell edges rather than the dotted shape FAs on the 24/24 µm # microtopography (Figure 6d). The combined landscape of 24/33 µm # structures led to cells aligning mostly to the 24 µm step creating (interrupted) ridges (Figure 5c) by bridging the top of the exposed inter-grid space (Figure 6a,b,d-along xz). The FAs aligned to the portions of the 24 µm step reveal ridges, as in the case of the 24 µm || structures (Figure 6b). Cells also were able to connect to the 33 µm step generated grooves (Figure 6c,d-xz), similar to the behavior on the 24/24 µm # (Figure 6c,d-yz).

### 3.4. Human Mesenchymal Stem Cell Proliferation onto Microtextured Zirconia

**Figure 7:**
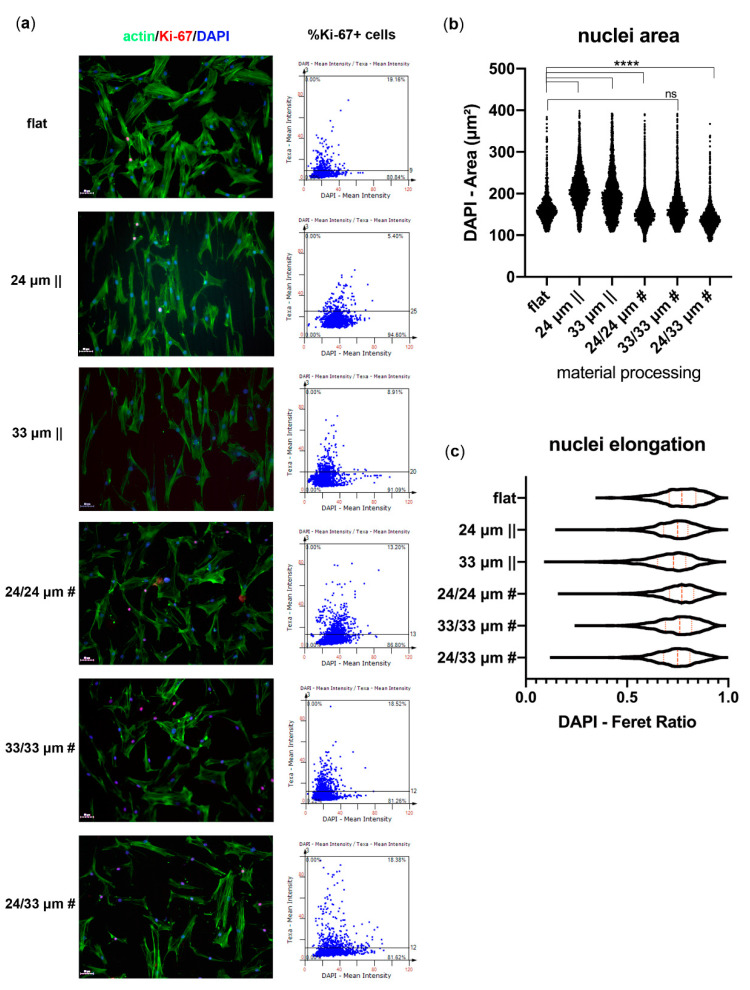
Proliferation of human mesenchymal stem cells (hMSCs) onto Zirconia microtopographies. Immunofluorescence microscopy images of Ki-67^+^ cell nuclei, and their corresponding image cytometry scatter plot quantification (**a**) at 3 days post-seeding onto the tested biomaterials. Nuclei area (**b**) and elongation (**c**) were analyzed for cell interaction with each of the structured Zirconia substratum. The fluorescence images (**a**) were obtained upon labeling with antibodies for Ki-67 (red) and staining of actin filaments with Alexa Fluor 488-Phalloidin (green) and nuclei with Hoechst (blue). Image acquisition was performed using the TissueFAXS iPlus automatic system. Data represent mean ± S.E.M., **** *p* < 0.0001.

To assess human mesenchymal stem cell (hMSC) proliferation on the microtextured Zirconia substrates, we next quantified the percentage of Ki-67^+^ nuclei at three days post seeding, using image cytometry (Figure 7a). Results showed cells grown on anisotropic patterns decreased their proliferative activity (5.40% and 8.91% Ki-67^+^ nuclei, respectively), compared to cells on the flat control (19.16% Ki-67^+^ nuclei). However, hMSCs grown on the proposed isotropic patterns generally maintained their proliferative ability (~18% for 33/33 µm # and 24/33 µm #), with a slight decrease observed for the cells grown onto 24/24 µm # structures (13.20% Ki-67^+^ nuclei). Most probably, the 33/33 µm # and 24/33 µm # isotropic patterns allowed cell-to-cell communication due to improved spreading (Figure 5a,b; Figure 7a) and to less restricted directionality (Figure 5d). The communication through cell-cell contacts may further allow paracrine signaling activation and, consequently, retain proliferative activity [68].

We next determined nuclei area and elongation tendencies based on the fluorescent DAPI staining (Figure 7b,c). Our results showed a significant increase in the nuclei area of hMSCs grown onto the isotropic patterns, while a statistically significant decrease was observed for the anisotropic 24/24 µm # and 24/33 µm # structures (Figure 7b), compared to the flat control. No significant difference was observed between the 33/33 µm # processed substrate and control. Calculation of the DAPI Feret ratio parameter provided evidence (Figure 7c) of a slight decrease in the aspect ratio of nuclei grown on 24 µm ||, 33 µm ||, and 24/33 µm # substrates (median Feret ratio 0.75), while the symmetric 24/24 µm # and 33/33 µm # anisotropic patterns presented largely the same shape as the control (median Feret ratio 0.8). These data indicate an elongation tendency for hMSC nuclei grown on anisotropic or asymmetric isotropic Zirconia substrates, which correlate with an increased nuclei area only for the anisotropic samples (Figure 7b). The 24/33 µm # substrates induce nuclei downsizing through limiting their area from spreading across the dominant cell guidance axis (24 µm || steps).

### 3.5. Osteogenic Differentiation onto Microtextured Zirconia

**Figure 8:**
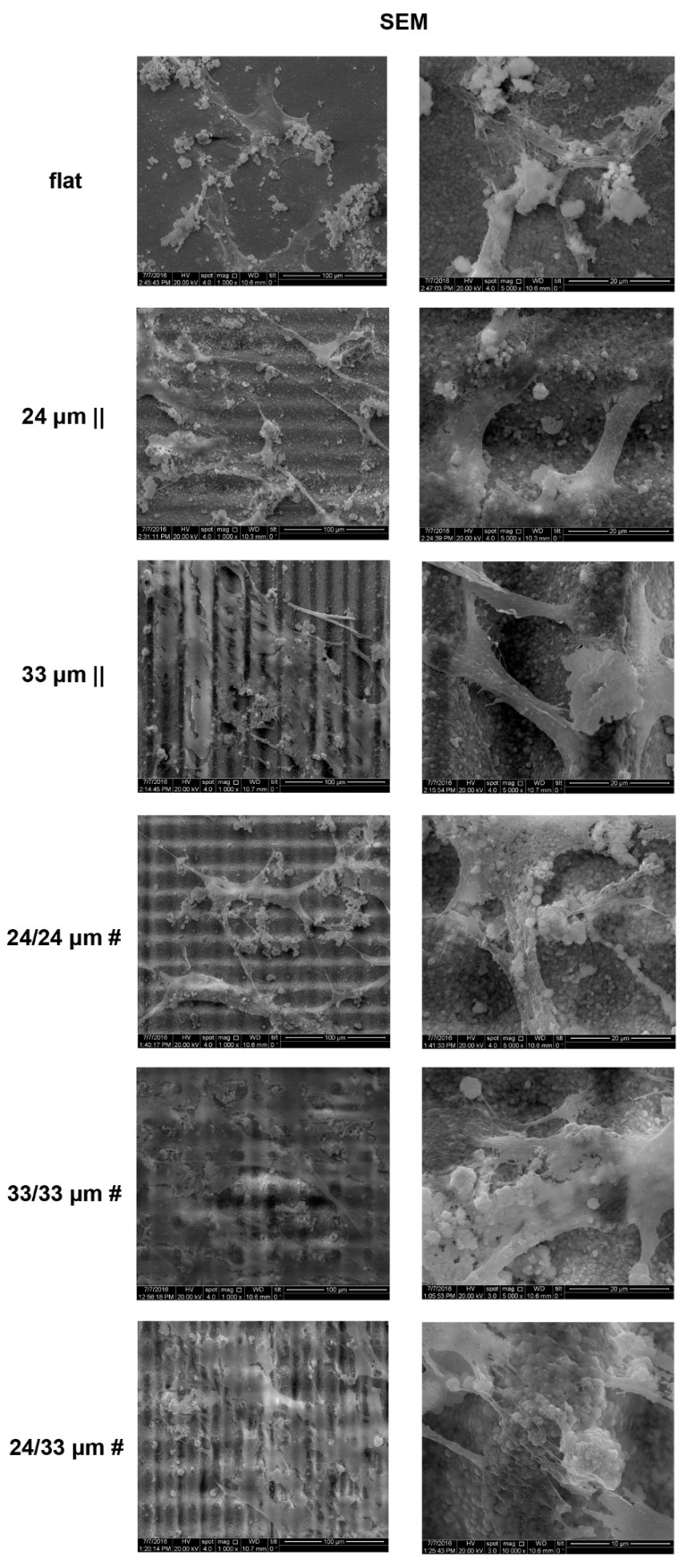
Cell morphology analysis upon osteogenic differentiation of human mesenchymal stem cells (hMSCs) onto Zirconia microtopographies. Scanning electron microscopy (SEM) images of cells cultured for 28 days on Zirconia bio-interfaces in osteoinductive media conditions. Low- and high-magnification SEM images are presented representing each of the structured Zirconia substratum.

To investigate human mesenchymal stem cell hMSC differentiation to osteoblasts on each of the microtextured Zirconia substrates, we employed scanning electron microscopy (SEM), Alizarin Red staining, immunofluorescence microscopy and EDAX. First, the SEM analysis performed at 28 days post-osteoinduction revealed differences in surface coverage by the in vitro differentiated cells between tested substrates. Cells grown and differentiated on the flat and 24 µm || processed Zirconia formed superficially attached clusters, while those on the 33 µm || and on the isotropic patterns provided better spreading cues (Figure 8, Figure S3). The distribution of mineralized clusters was, however, zonal on the 33 µm ||, as well as on the 24/24 µm # and 24/33 µm #, while osteoblasts differentiated onto the 33/33 µm # processed sample showed the most uniform surface coverage (Figure S3). The SEM images revealed that after 28 days in culture, cells form diagonal bridges over the microgrooves thereby linking lines/ridges, without having intimate contact with the microtopographies’ deep structures (Figure 8). However, osteoblasts generated onto isotropic patterns form network-like meshes more resembling the desired in vivo tissue-biomaterial interaction (Figure 8); the 24/24 µm # structure support thin interconnecting cell bodies, while the 33/33 µm # and 24/33 µm # topographies provide guidance for the formation of more stably spread interconnected cell clusters (Figure 8, Figure S3).

**Figure 9:**
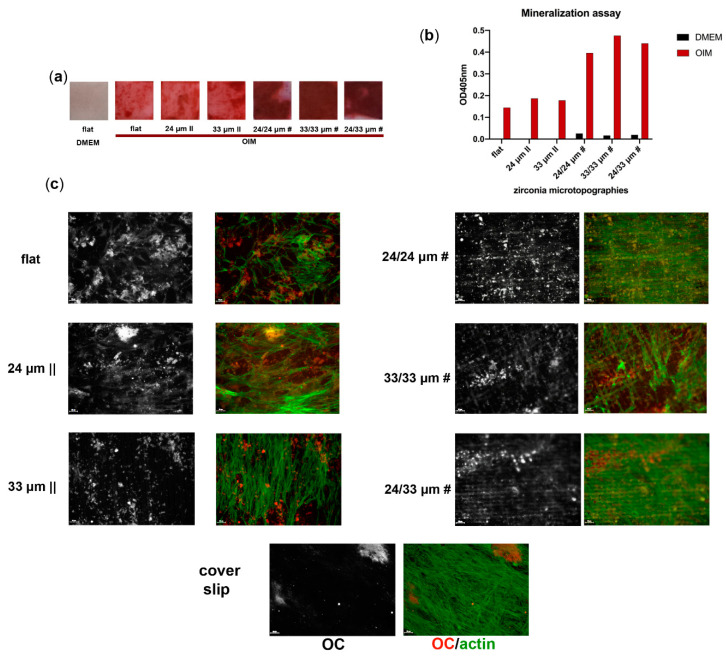
Osteogenic differentiation of human mesenchymal stem cells (hMSCs) onto Zirconia microtopographies. Alizarin Red staining (**a**) and quantification (**b**) for hMSCs cultured for 28 days on Zirconia bio-interfaces in osteoinductive media conditions (OIM) versus control (DMEM). Immunofluorescence images of cells expressing the osteoblast marker osteocalcin at the end of the differentiation process (**c**). The fluorescence images (**c**) were obtained upon labeling with antibodies for osteocalcin (red) and staining of actin filaments with Alexa Fluor 488-Phalloidin (green). Image acquisition was performed using the TissueFAXS iPlus automatic system.

To determine the impact of Zirconia microtexturing on osteogenic mineralization, we next stained cells with Alizarin Red to assess Calcium (Ca^2+^) ion deposition during differentiation. Our results indicate that hMSC differentiation efficiency on anisotropic patterns is similar to that obtained on the flat control, while the osteoblast grown on the isotropic Zirconia surfaces generate enhanced mineralization (Figure 9a,b), correlated with the observed cell coverage (Figure 8, Figure S3). The fluorescence microscopy results show a disposition of cells along with the anisotropic microgroove patterns, while cells on the isotropic substrates developed a grided mesh-like structure, compared to the random orientation of cells on the flat Zirconia and standard microscopy glass cover slip (Figure 9c, actin filaments in green), thereby complementing the SEM analysis data. The osteocalcin (OC) protein expression deposited by the differentiating cells was detectable on all substrates at the same 28-day time point (Figure 9c, OC in red). A trenched OC matrix pattern was evident on the anisotropic Zirconia substrates; a dotted OC pattern was present zonally on the 33/33 µm # and 24/33 µm # samples and, more uniformly, on the 24/24 µm # (Figure 9c, OC).

**Figure 10:**
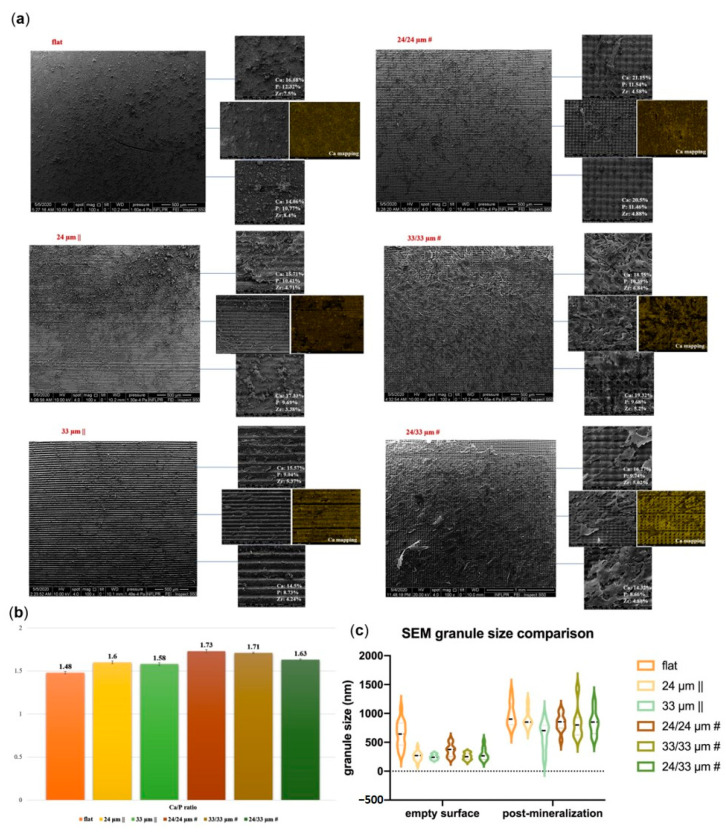
Chemical and physical analysis of material surfaces after human mesenchymal stem cell hMSC osteogenic differentiation. (**a**) Scanning electron microscopy (SEM) images of hMSCs cultured for 28 days on Zirconia bio-interfaces and their corresponding EDAX measurements. Low- and high-magnification SEM images are presented representing each of the structured zirconia substratum. The atomic percentage chemical composition from the EDAX is presented along with the representative SEM magnified images. EDAX area mappings also were performed to detect Ca element. (**b**) Ca/P ratio at the end of the differentiation process (28 days). (**c**) Granule size assessment on SEM images before and after mineralization.

Calcium nodule formation was observed in all the samples (Figure 10a), with differences in their conglomeration and distribution depending on the topography type. The density and uniform distribution of the nodules, together with Ca mapping, are underlying and confirming the mineralization tendency observed in Figure 9. The calculated Calcium/Phosphates Ca/P ratio for Zirconia bio-interfaces was within the range of 1.48–1.73 (Figure 10b). The values for grided patterns 24/24 µm # (1.73), 33/33 µm # (1.71), and 24/33 µm # (1.63) are close to that previously reported by Bose et al. [70] for typical adult human calcified tissues (bone and enamel, respectively). The increase in surface granulation observed in Figure 10c, especially for the laser-processed surfaces, is an indication that Zirconia-processed materials are serving as mineralization nucleation centers.

Overall, our analyses show that the Zirconia’s grid-like laser processing provides the necessary cues to obtain increased osteogenic differentiation, with emphasis on the 33/33 µm # structure that allows an optimal 1.71 Ca/P ratio, as well as bone cell density, both equally crucial for further application in prosthetics.

## 4. Conclusions

Next-generation biomaterials are based on the synergy and correlation between material surface features and cell responses in terms of control of cell adhesion, proliferation, growth, and differentiation. Here, bioinstructive micro-nano hierarchically structured Zirconia ceramic surfaces for guiding and stimulating osteogenic responses were proposed as a biomimicry approach. The proposed solution relied on the innovative design of surfaces, requiring the use of a femtosecond laser to microtexture the ceramic into tailored architectures like anisotropic microgrooves/ridges, as well as isotropic pillar-like structures for providing human mesenchymal stem cell (hMSC) adhesion, spatial orientation, and mechanical stimuli for differentiation into bio-mineralized structures and tissue-like constructs. Our results demonstrated an improved osteogenic phenotype of hMSCs when grown onto isotropic grid/pillar-like patterns, with enhanced coverage and a Ca/P ratio of 33/33 µm # processed Zirconia. This has direct implications for bone-anchored hearing aid (BAHA) prosthetic development, where the replacement of Titanium with Zirconia would offer esthetic advantages and optimize new substrates for bone tissue engineering purposes, using a laser-based technology that provides optimal control and reproducibility. Further work needs to be done to optimize material functionality regarding its auditory wave propagation capacity to be amenable to BAHA prosthetic use.

## Figures and Tables

**Table 1:** Surface energy values of the textured areas.

Samples Type		γ_d	γ_p	γ_t
Non-Irradiated Control	Flat	26.35	5.17	31.52
Anisotropic patterns	24 µm ||	47.61	12.63	60.24
	33 µm ||	49.29	8.77	58.06
Isotropic patterns	24/24 µm#	47.43	17.75	65.18
	33/33 µm #	48.75	14.63	63.38
	24/33 µm #	46.73	13.16	59.89

## Supplementary Materials

The following are available online at https://www.mdpi.com/2079-4991/10/12/2465/s1, Figure S1: SEM and AFM images of the lasers textured surfaces, depicting profiles and depths, Figure S2: Early attachment of hMSCs onto Zirconia microtopografies. Immunofluorescence microscopy depicting hMSCs onto each of the structured Zirconia substratum (a) upon labeling with antibodies for vinculin (red), and staining of actin filaments with Alexa Fluor 488-Phalloidin (green) and nuclei with Hoechst (blue) (scale bar = 50 µm). Representative cells are showed in detail (b) to depict the vinculin-labeled focal adhesions (scale bar = 20 µm). Image acquisition was performed using the TissueFAXS iPlus automatic system, Figure S3. Cell morphology and mineralization granules analysis upon osteogenic differentiation of hMSCs onto Zirconia microtopografies. SEM images of cells cultured for 28 days on Zirconia bio-interfaces in osteoinductive media conditions. Low- and high-magnification SEM images are presented representing each of the structured Zirconia substratum along with granule size measurements before and after hMSCs culture and differentiation. Table S1: Average surfaces parameters as measured by profilometry, SEM and AFM.
