# Supplementary material for: Bioinstructive Micro-Nanotextured Zirconia Ceramic Interfaces for Guiding and Stimulating an Osteogenic Response In Vitro
Source: Nanomaterials (Basel). 2020 Dec 9;10(12):2465. doi: 10.3390/nano10122465 (PMC7764817; doi:10.3390/nano10122465)
Supplement: Supplementary file 1 [file nanomaterials-10-02465-s001.pdf]

# Supplementary Materials: Bioinstructive Micro-Nanotextured Zirconia Ceramic Interfaces for Guiding and Stimulating an Osteogenic Response In Vitro

**Livia Elena Sima [1], Anca Bonciu [2,3], Madalina Baciu [4], Iulia Anghel [2], Luminita Nicoleta Dumitrescu [2], Laurentiu Rusen [2,*] and Valentina Dinca [2,5,*]**

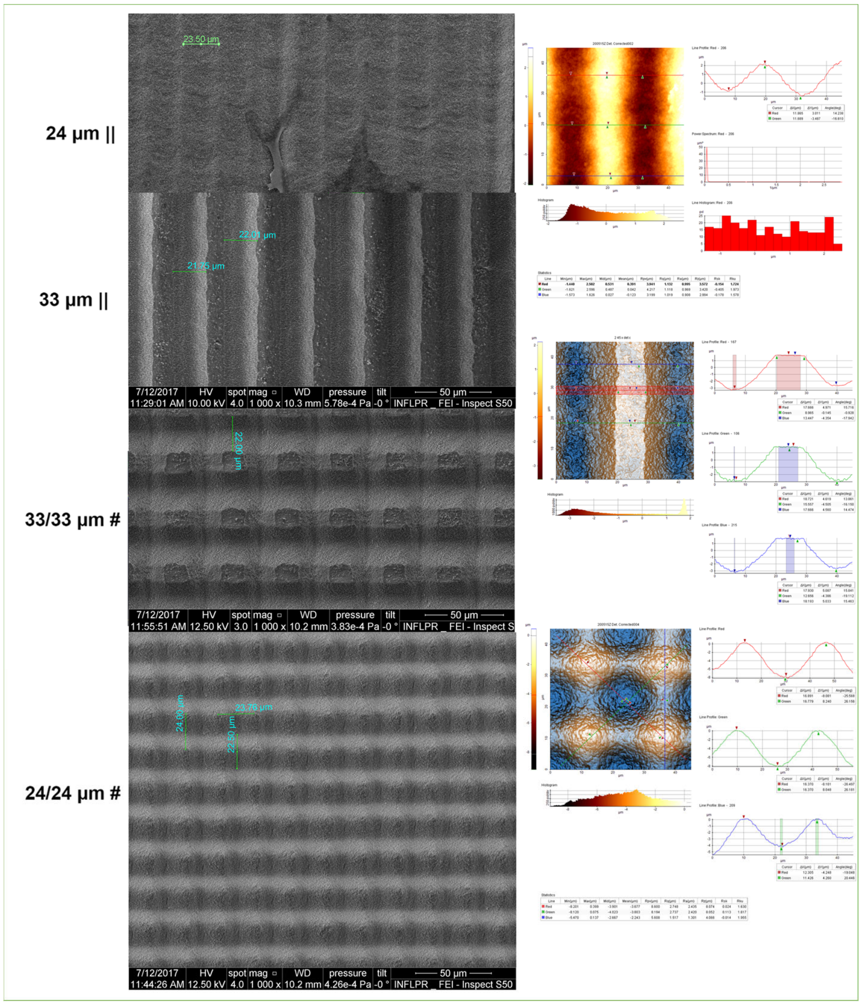


**Figure S1.** SEM and AFM images of the lasers textured surfaces, depicting profiles and depths.

**Table S1.** Average surfaces parameters as measured by profilometry, SEM and AFM.

| **Sample type** | **Sample name** | **Topography** | **Rq 3d [µm]** | **Rq profil [µm]** | **Center-to-center 2 point distance-lenghts** | **Average trench height** | **Average top width** |
|---|---|---|---|---|---|---|---|
| non-irradiated control | flat | | 0.123 (± 0.03 ) | 0.22 (± 0.10 ) | NA | NA | NA |
| anisotropic structures | 24 µm ǀǀ | wavy ridges | 2(± 0.042 ) | 3.03 (± 0.16) | 23.3 (± 0.46) | 3.39 (±0.797 ) | 0.99 (± 0.22 ) |
| | 33 µm ǀǀ | rectangular ridge | 1.4(± 0.15) | 4.08 (± 0.28 ) | 34.87 (± 0.19 ) | 4.3 (± 0.01 ) | 10 (± 0.89) |
| isotropic structures | 24/24 µm # | wavy grating | 2.3(± 0.2 ) | 3.14 (± 0.44) | 23.5 (± 0.6 ) | 3.46 (± 0.025) | 0.95 (± 0.1) |
| | 33/33 µm # | square pillar | 2.8(± 0.18) | 2.48 (± 0.25 ) | 35.2 (± 0.63 ) | 4.53 (± 0.02) | 11.09 (± 0.68) |
| | 24/33 µm # | square pillar | 2 | 1.92 (± 0.4) | 34.8 (± 0.19 ) and 23.3 (± 0.55 ) | 4.37 (± 0. 31) | 5.14 (± 0.87) |

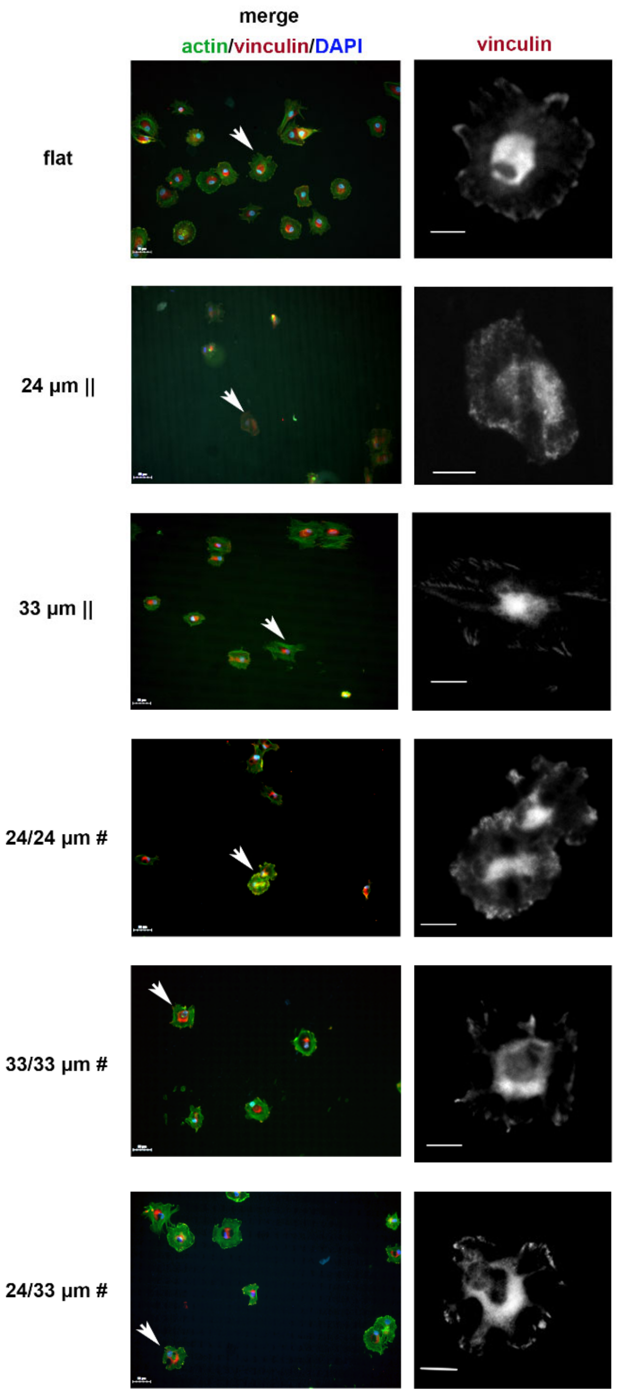


**Figure S2.** Early attachment of hMSCs onto Zirconia microtopografies. Immunofluorescence microscopy depicting hMSCs onto each of the structured Zirconia substratum (**a**) upon labeling with antibodies for vinculin (red), and staining of actin filaments with Alexa Fluor 488-Phalloidin (green) and nuclei with Hoechst (blue) (scale bar = 50 μm). Representative cells are showed in detail (**b**) to depict the vinculin-labeled focal adhesions (scale bar = 20 μm). Image acquisition was performed using the TissueFAXS iPlus automatic system.

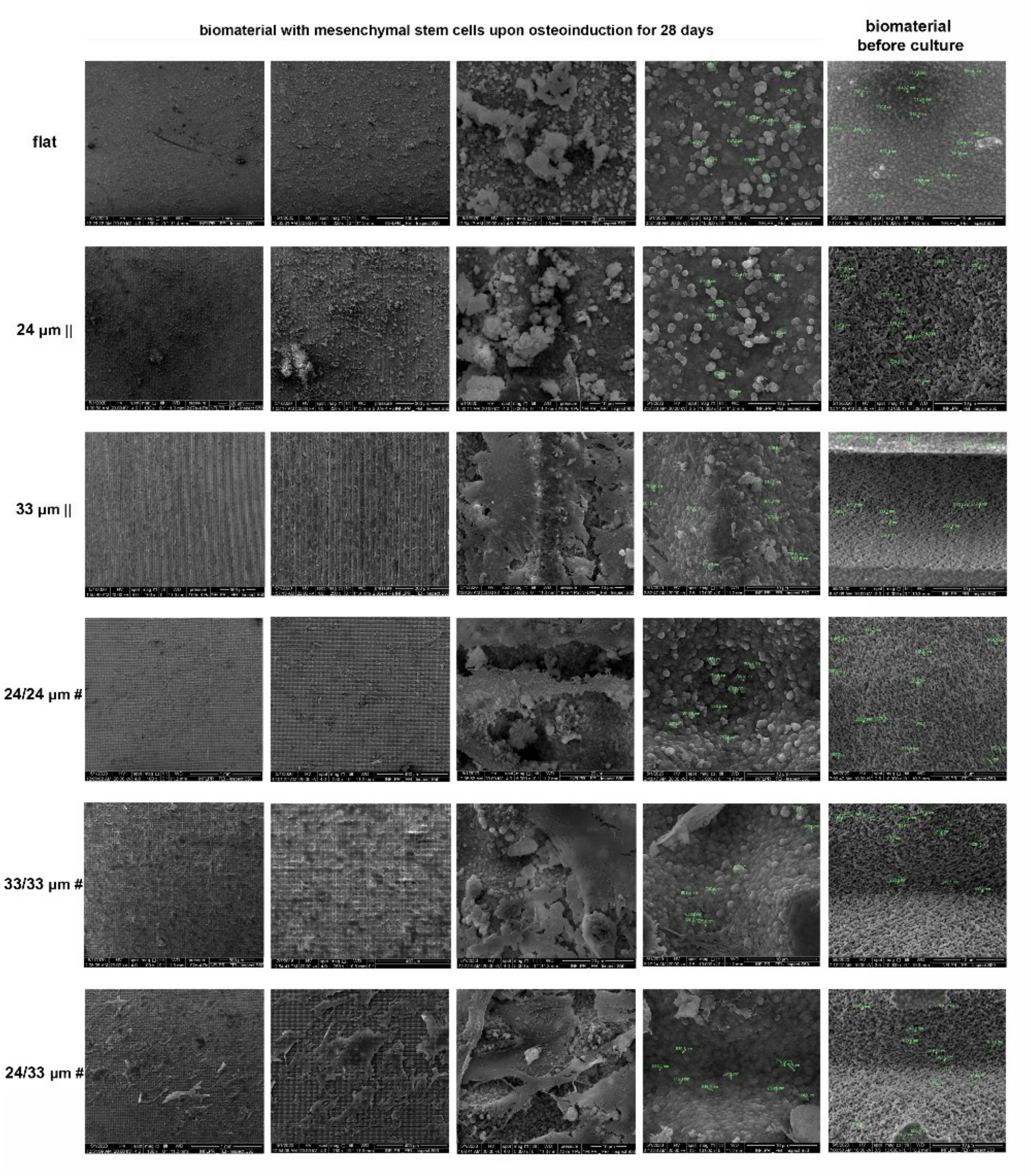


**Figure S3.** Cell morphology and mineralization granules analysis upon osteogenic differentiation of hMSCs onto Zirconia microtopografies. SEM images of cells cultured for 28 days on Zirconia bio-interfaces in osteoinductive media conditions. Low- and high-magnification SEM images are presented representing each of the structured Zirconia substratum along with granule size measurements before and after hMSCs culture and differentiation.
